# Typho-Malarial Fever

**Published:** 1866-11

**Authors:** Wm. H. Veatch

**Affiliations:** Pawnee, Sangamon Co., Ill.


ARTILE XXXIX.

### TYPHO-MALARIAL FEVER.

By WM. H. VEATCH, M.D., Pawnee, Sangamon Co., Ill.

Read to the Illinois State Medical Society, June, 1866.

I do not presume that I am the only member of this Society in whose practice has occurred cases of the disease that I am now about to delineate; neither would I arrogate to myself a preëminence in its successful treatment; nor would I say that I desire to present anything to the Society but my own humble experience in the diagnosis and treatment of a disease that, to say the least, has proven itself quite formidable, and one whose diagnostic signs were, in the first cases, so obscure that some time had elapsed, and several cases had been treated by myself and others, before an appropriate appellation could be given it; and even now, some good physicians are disposed to call in question the propriety of applying the name that I think the most appropriate. But as names are applied to diseases, corresponding to their nature and character, and as I believe the profession will sustain me in the name best calculated to convey to the mind of the medical student a correct notion of the nature and character of the disease he is trying to understand, I must be allowed to persist in calling the malady under consideration an epidemic of Typho-Malarial Fever.

A disease of a febrile character, which is manifested by a condition of the human system in which coma is a prominent symptom, we would call typhus or typhoid; and one whose signs manifest all the characteristics of a disease originating from exposure to causes generally known to produce hepatic forms of disease, we would denominate malarial fever; and a fever that partakes of the nature and character of both the above forms of disease, science would dictate a name corresponding in significance to the nature and character of the double form the disease has assumed.

When a disease makes its appearance in a community and affects those only of a certain district, or is confined in its ravages to a certain class, we call it endemic. But if it prevails generally, we call it epidemic. And so, throughout the whole catalogue of maladies to which flesh is heir, we apply names corresponding to their nature and character.

The term *typho-malarial* carries with it an idea of a double form of disease, just the idea that is desired to be conveyed when speaking of the epidemic now under consideration, for almost all cases of fever occurring under my notice during the autumn of 1864 and throughout the year of 1865, partook more or less of this double character. But without farther discussing the propriety of the name at this point, I will proceed to a partial sketch of its history.

This fever made its appearance epidemically in the vicinity of Pawnee, Sangamon Co., Illinois, during the autumn of 1864, and, as the season advanced, its character was more and more developed, until the beginning of the winter, when it became so malignant that the mortality was somewhat alarming; but towards spring its malignancy abated to some extent. As the warm weather of 1865 set in, its virulence increased, and throughout the whole season was very severe, although the mortality was not so great, owing, perhaps, to the fact that its nature had become better understood, and, consequently, a more judicious application of remedies.

The first cases coming under my care, had all the appearances of a common remittent in its ordinary form of chills—thirst, transient pains through the body, aching of the back and limbs, tongue covered with a white or yellowish coating, sickness of the stomach, restlessness, and a remitting fever—but after from three to seven days of these symptoms, a decided typhus character was assumed, and the patient would pass rapidly into a condition not unlike the third stage of typhoid fever, but by the time the third stage of typhoid should have made its appearance the patient would either have passed through the apparent third stage of typhoid and convalescence be established, or else he would have sunk into collapse and died, on or before the fifteenth day.

There was one case so peculiar in its progress, that I must delineate its history:—A widowed lady, of 35 years of age, delicate constitution, melancholy disposition, bilious temperament, was taken sick November 26th, 1864, with all the signs of an ordinary bilious remittent, as above described. 27th. A remission, followed by a recurrenee of chills, and a more aggravated character of fever. 28th. Remission very slight, no chills, tongue heavily coated and dry, great thirst, extremely restless and manifests a preternatural strength, pulse full and bounding, bowels moving, bilious evacuations, skin pale and covered with a heavy perspiration. 29th. No sleep, notwithstanding the free administration of opiates during the night; pulse 110, full; tongue dry and more brownish; throat shows signs of congestion; soft palate and tonsils have a dark red somewhat puffed appearance. 30th. Countenance anxious, very pale; pulse 120, and small; all other signs as on the day previous. Dec. 1st. Notwithstanding the free use of alcoholic stimulants, a tendency to collapse continued; tongue almost black; pulse 130, very small, and easily compressed; throat shows signs of an exudation of blood. Coma came on by degrees, and she sank on the 2d, with all the signs of collapse of typhus. After death, petechiæ made their appearance over the chest, the abdomen and back being covered with small spots and blotches, from the size of a pea to that of a silver half-dollar, irregularly distributed over those parts, of a dust-brown color, some of them being almost bronzed. No *post mortem* allowed.

Other cases, similar to the above in the beginning, would run on to the eighth or ninth day without any symptoms of a typhus or typhoid character, and, when looking for a convalescence to be fully established, a recurrence of the chills would take place, followed by an aggravation of all febrile signs, and in a day or two petechiæ make their appearance, delirium set in, congestion take hold of the soft palate and tonsils, heavy perspiration cover the surface of the body, diarrhœa would ensue, collapse and death by the fifteenth day; or, about the fourteenth day, would arouse, as from a profound slumber, and convalescence would be at hand. Other cases would run the course of an ordinary remittent, and, when about to recover, to all appearances, congestion would seize upon some of the vital organs, and soon it would be carried through the whole system and the patient sink under symptoms not unlike those of the pernicious intermittents of the old writers, or, as we usually term it, congestive chill.

All cases under my care recovered if they passed over the fifteenth day, except one, and I think most of the fatal cases proved so on or before the fifteenth day.

A few cases relapsed and died, I have been told, after convalescence had been fully established, but none of that class came under my notice. Yet another class of cases was occasionally met with, not unlike the beginning of the ordinary typhoid of this climate. The patient would feel indisposed for many days before the real attack would come on, indisposition commenced by general lassitude, depraved appetite, and, after some days, headache would come on by degrees, bowels either completely constipated or a watery diarrhœa of some days standing, referable, perhaps, to a dose of pills or other cathartic medicines taken at the beginning of the indisposition, and after from seven to fifteen days of simple indisposition, slight chills, with aching of the back and limbs, would indicate the coming on of a malarious form of disease, but the tongue showing a heavy brown coating down the centre, the tip and edges having a glossy red appearance, and a continued feverish condition of the whole system. After these symptoms had lasted from three to seven days, wild delirium set in, and the patient was said to have typhoid fever. Soon, the throat showed signs of congestion, and often the glands became tumefied and inflamed, and sometimes even suppurate, but more often an ulceration, not unlike that of diphtheria, would spread over the inner surface of the throat, the alvine evacuations having a muddy water appearance, and in a few hours, or a day or two at farthest, the patient would sink into a profound stupor, which usually terminated in death from seven to fifteen days after the appearance of the first chills; but when stupor did not come on, the patient would pass through a course of typhoid fever, and recover after a course of from fifteen to forty-two days. Not a few cases came under my care, the first symptom of which was the ulceration of the throat, the attendant fever being of a typhus character.

The eruption did not make its appearance at any regular period of the disease, sometimes coming on as early as the sixth or seventh day, and in other cases not until the eighteenth or twentieth, and one case had no signs of the eruption until the twenty-sixth day. In a few cases it could not be seen at all, and in those cases where the eruption was not to be found at any period during the fever, the symptoms were as urgent as in those having the greatest amount of it.

In all cases, the abdominal region presented a field for much anxious thought. I do not remember of treating a case in which there was not more or less trouble with the abdominal viscera; at times, however, it showed no points of interest until after the fifteenth or eighteenth day, when suddenly the abdomen became distended and tender, the countenance anxious and pale, great restlessness, alvine evacuations muddy and more abundant, and without speedy relief must sink into collapse and die.

There is one symptom in this affection that in most cases was a very prominent one, and one that gave me more trouble than any one symptom of the disease, and one, too, calculated to throw the practitioner off his guard unless he is acquainted with it, and, in my opinion, it is one of the best prognostic signs belonging to this form of disease. I have reference to a constant warm perspiration, often beginning with the onset of the disease and continuing throughout its whole course, and those cases in which the perspiration was the most persistent were also the most obstinate to control, and a greater number proved fatal, in the progress of which the perspiration could not be checked, than in any other class of cases, which gave rise to the expression of the above opinion, that the state of the diaphoresis was one of the very best prognostic signs of the disease. In all cases where the perspiration ran on to a great degree before it could be checked, the bowels became the agent of prostration as soon as the perspiration ceased. It appeared that the work of prostration must be carried on through some channel until the virus of the disease had spent its force in the system and lost its power for harm, or death cut short the debilitating process.

This scourge had all the characteristics of an epidemic form of disease, and in some instances showed signs of contagion, as, frequently, nurses would be subjected to severe attacks; but in all cases of apparent contagion the disease could be accounted for by other existing causes, such as the general epidemic influence to which all were more or less exposed, and the same depressing influences to which those were exposed who had no opportunity whatever to have it communicated to them by contagion, and the great number of such cases would seem to preclude the idea of contagion altogether.

There does not appear to be anything peculiar in the surroundings of this vicinity calculated to lead to a solution of the cause or causes of so malignant a form of disease as this has proven itself to be. The only matter worthy of consideration in this respect is the fact, that all over this county is generated the elements of a koino miasma which is generally admitted to be the predisposing cause of hepatic forms of disease. And, in connection with this, it will be remembered that during the two years of the prevalence of this disease, the earth was completely covered with all kinds of vegetable growths belonging to this climate, the people subsisting to a great extent on them.

The county is in a pretty high state of cultivation, and has a sufficient amount of undulation to prevent, to a great extent, the stagnation of water. Many small streams make their way through the prairies, emptying their waters into the larger timbered streams, the water being supplied from numberless small springs opening along their banks, constituting circumscribed marshes, from which arises the principal part of the koino miasma. But these marshes do not appear to be sufficiently extensive to have in them the amount of malignancy that this epidemic carried with it; neither do the habits of the people appear to have anything to do in the production of the disease. So far as the generation of an idio-miasma is concerned, the people almost all live in light wooden tenements, and but few of them have cellars.

Then our attention must be directed to some other agent for a full solution of the causes of the affection. I have already said that during the two years of the prevalence of the epidemic we had an abundant crop of all kinds of vegetation, and a great amount of the people's food consisted in vegetable matter. Now, it has been pretty fully demonstrated, that in animals subsisting on vegetable substances a greater amount of carbonic acid is retained in the blood than in those which subsist on animal food; and, according to DALTON, a less proportion of oxygen is retained in the system and a greater amount thrown back by exhalation in the carnivora than in the herbivora, in the proportion of about three to one. We must, therefore conclude that in those persons whose principal subsistence has been vegetable, that the blood is minus the proper amount of life principle, the oxygen, and plus the carbonic acid; consequently the blood is in precisely the same condition of those persons who are being exposed to the idio-miasma of densely populated cities, crowded hospitals, and prisons, where many persons are crowded together, in small, illy-ventilated tenements, which has been considered by the best authors as one of the causes of typhus and typhoid fevers. What chemical changes these miasmata produce in the blood I cannot say; but one important fact is patent to the most casual observer, *viz.*:—That these miasmatic influences exert a very great power of man's functional organization, and the two distinct influences acting on man's vital apparatus at one and the same time must, of necessity, exert a twofold effect in producing disease, and much greater reason have we to ground this theory when we remember that the action of carbon in the blood produces delirium and, finally, coma, two of the most prominent symptoms in this affection. So, in this view of the matter, we find a solution to the great question of the causes producing the double form of disease, which also puts forever to rest the already exploded sophism, that two distinct causes cannot affect the system at one and the same time. These separate causes not only affect man at one and the same time, but, when each has had its specific effect in the system, they combine in producing an idio-miasma that not only affects the patient himself, but affects, also, those in attendance, thus supporting the popular notion of the contagiousness of the disease.

This epidemic had no respect for age, sex, or condition of life; the infant at the breast, the hoary-headed grandsire, and all the intermediate stages of life were subject to its ravages. But persons from fifteen to thirty years of age appeared to be more subject to it than the extremes of life; and males seemed to be more susceptible to its influences than females, owing, perhaps, to the greater amount of exposure to the causes.

All that is now left to be considered, is the principles of successful treatment. Now, in the treatment of any form of disease, it is proper, first, to consider the cause or causes producing the disease, and, if possible, remove them. But when the causes are so obscure that they cannot be definitely ascertained, we must be guided into a correct course of treatment by closely observing the effect of those causes, and if, happily, we succeed in counteracting them, we are practising from a correct theory, a rational standpoint; and if I should be mistaken as to the specific causes producing the disease, I cannot be mistaken in the effects produced by them. I have just shown that the vital powers of man are vehemently attacked by the venom of the disease; that delirium and coma are prominent symptoms in its course; and that these symptoms probably depend on a carbonized condition of the blood, for every characteristic of the disease proves a general depraved condition of the vital fluid, and, consequently, an inactive condition of all the vital organs.

A treatment, therefore, that will give renewed energy to the wasting vital powers of the system, a treatment that will increase the vital principles in the blood, give energy to the dormant functions of nature's complicated apparatus, arouse the latent powers of absorption and assimilation, bring back a unity of action between the several organs whose functions are so materially disturbed by the action of a blood poison; in a word, a treatment whose object it is to build up trodden down tissue and act in a direct opposite course to that taken by the disease, will, of all others, in my opinion, be the most eminently successfully. If a blood poison produces debility and prostration, it is the first duty of the physician to administer agents that will impart tone and energy. If a blood poison produces a carbonized condition of the blood, it is the duty of the practitioner to have recourse to those remedial agents that will impart oxygen to the vital fluid of life and thereby generate a more energetic electric current through the whole system, that all parts of the vital domain may have increased energy to cast off morbific agents, and that each part may have power to perform the important duties respectively assigned them by nature.

After we have determined upon a rational course to be pursued, another very important question arises:—Out of the whole catalogue of remedial agents at our command, which are to be chosen, and how are they to be employed, that we may best accomplish the end in view? Shall we by purgatives, in the onset of the disease, prepare our patients for stimulants and tonics in the latter stages? I answer, no!

Secondly. Shall we administer emetics and powerful diaphoretics, that we may eliminate poison from the blood, and thus have a rapid reaction of the vital powers to cast off morbific agents? I answer, never!

Thirdly. Must we, by a persistent course of mercury, excite secretion to such an extent that nature, by her own action, may attempt to build up broken down tissue? My answer to this, also, must be emphatically in the negative.

First. By the administration of purgatives in the onset of the disease, we relax the coating of the stomach and bowels, and cause a pouring out of the fluids of the blood into their cavity, consequently, a more or less severe diarrhœa, characterized by the rice-water or soapsuds evacuations, and, instead of being a benefit, they only prepare those organs to cast off remedial agents in after treatment, by which we desire to build up the strength of the patient in arousing absorption and assimilation.

Second. By the administration of emetics and powerful diaphoretics, we only open up the channels through which the supplies of the great storehouse of nature will be carried off and squandered when the disease has set complete seige to the citadel of life.

Third. Mercury is known to be a powerful defibrinizer of the blood, and, when steadily administered, not only diminishes that property of the vital fluid, but also becomes a glandular irritant, by exciting secretion when there is nothing but the principles of depravity to be secreted. We thus cause an unnecessary routine of action in the secreting organs, and instead of building up the works of defence, we break down our already weakened fortifications. We must, therefore, conclude that the whole character of mercury, as a remedy in this disease, is an unmitigated evil, and if we would save our patients a great degree of prostration, we must give neither emetics, powerful diaphoretics, nor drastic purgatives; and above all, would I warn you against the use of mercury, to excite secretion, until you know that you have something more than poison to be acted on.

For fear that my position may be misunderstood on this point, I will here say, that I am a great admirer of the above named remedies in their proper and legitimate places, but I must be allowed to say that I do not believe their places exist in the treatment of typho-malarial fever. Our whole plan must be, as I have already indicated, to build up the system by promoting absorption and assimilation, and not arouse secretion too high until we have something in the blood from which to make tissue.

Now that I have pointed out the general principles on which a treatment is to be conducted, in my own humble judgement, I will proceed to the specification of such articles as, in my opinion, will be best calculated to accomplish the end in view.

When we find a patient laboring under obstinate costiveness, we should move the bowels by a mild enema of castile soap and molasses; this will cleanse the lower bowels and allow the contents of the stomach to pass down without irritation, and thus avoid the obstinate diarrhœa so often met with after the administration of calomel, blue mass, colocynth, jalap, or any other drastic purgatives. After the action of the enema, we should direct the patient to have a mild opiate, to promote rest; remove all sources of anxiety from the mind; and allow him to drink freely of a saturated solution of either chlorate or the acetate of potassium. This will oxygenize the blood, cause a renewal of the electric current through the system, prevent the decay of tissue, and also promote absorption and assimilation. After this course has been pursued from twenty-four to forty-eight hours, owing to the condition of the patient, we may add to the treatment a pill composed of chenoidine and ipecacuanha, two grains of the former to one of the latter, made into mass with the oil of black pepper—one to be given every two or three hours; and a Dover's powder at night, where there is not too much delirium, and, where the opiate cannot be used, the extract of hyoscyamus may be combined with the pill with great advantage. This comprises about all the constitutional treatment necessary in ordinary cases. The body of the patient should be washed twice a-day in soapsuds at first, and in the latter stages may be added to the wash alcoholic spirits, to give tone to the integuments of the skin. Applications of sinapisms and fomentations, for local irritations and complications, must not be neglected, particularly when the bowels become distended and tender, and accompanied by diarrhœa, which is almost universally the case at some period of the disease. If diarrhœa set in, which is sure to be the case if purgatives have been freely used in the beginning, astringents are to be used both by the mouth and rectum. If delirium supervene, cold water or ice to the head. If heavy perspiration comes on, the dilute sulphuric acid must be freely used. Add to this treatment a proper course of nourishment, freely given, and in such manner that the greatest amount can be taken in the smallest compass, such as beef-tea, milk-punch, egg-nog, etc. Close attention to the condition of the bowels is one of the most important matters connected with the whole treatment, and by allowing the bowels to be too freely moved in the beginning, is, in my opinion, the cause of many cases proving fatal that would otherwise recover.

After first emptying the bowels by the use of a proper enema, they should not be moved again during the whole course of the fever; and if they should move spontaneously more than once in three days, astringents are indicated. In this position, I am aware that I will meet with opposition, and that too from some of my medical brethren for whose professional worth I have great respect. But as that opposition is founded on a mistaken notion of the correct theory of successful practice, I must be allowed to sustain the position by a statement of a few successful cases treated by myself, in proof of its correctness.

In my practice, I have demonstrated, to my own satisfaction, that the longer the bowels are held in check, the better for the strength of the patient; the more rapid the convalescence, the less liable to relapse; and from ulceration of the bowels, I consider the patient entirely exempt—a result so often met with where the bowels have been allowed to move too freely in the beginning. Of twenty-two cases treated by myself, those kept the longest without an action on the bowels made the most rapid convalescence; and, conversely, those that were allowed to be moved freely in the beginning of the fever were the most tardy and the hardest cases to control, other circumstances being equal. Of these twenty-two cases, eight were held twelve days; six were held fifteen days; five, sixteen days; and three were held eighteen days. Many others were held from six to eleven days, with the same comparative result.

I do not remember of but one case proving fatal after the bowels being held in check as long as three days, and that case was so completely prostrated from the effects of carbon in the blood, that, as soon as the bowels checked, the colliquative perspiration took the place of the diarrhœa, as the prostrating agent, and he died six days after the bowels had been moved.

There is, yet, only one prominent symptom, the treatment of which is to be considered, namely, the throat affection. Many were found to have ulceration of the throat among the first symptoms of the disease, and every case in which the persulphate of iron was used as a gargle, the ulceration was almost immediately healed; and in those cases where the throat only took on congestion, the persulphate of iron was the most efficacious of all applications. I have also used with great benefit, the muriated tincture of iron, but as that is so common among all classes of physicians, its special notice here is unnecessary. But as I have never seen the persulphate recommended in this affection, I beg leave to call special attention to it, both as a gargle and as an excellent remedy in case of ulceration of the bowels, in doses of from three to five grains every two hours. This article, in my hands, has proved to be one of the most efficacious I have ever used.

I cannot close this article, without expressing my preference for the use of the gargle over that of the probang, in the throat affection. I have seen many cases in which I am confident that the probang did as much harm as the remedy did good; and in all cases where it is practicable, I would advise the use of the gargle in preference. But in children, or in adults who are so delirious they cannot be made to understand, the probang will have to be resorted to, but then it should be used with care.

In conclusion, allow me to say, I have only attempted to give my own opinions, backed up by my own practice, regardless of authority or the expressions of opinion by other members of the profession, and I think the principles herein set forth can be fully sustained in practice.

